# The Double-Edged Role of Extracellular Vesicles in the Hallmarks of Aging

**DOI:** 10.3390/biom13010165

**Published:** 2023-01-13

**Authors:** Nekane Romero-García, Javier Huete-Acevedo, Cristina Mas-Bargues, Jorge Sanz-Ros, Mar Dromant, Consuelo Borrás

**Affiliations:** 1 Department of Anesthesiology and Surgical Trauma Intensive Care, Hospital Clinic Universitari Valencia, University of Valencia, 46010 Valencia, Spain; 2 Freshage Research Group, Department of Physiology, Faculty of Medicine, University of Valencia, Centro de Investigación Biomédica en Red Fragilidad y Envejecimiento Saludable-Instituto de Salud Carlos III (CIBERFES-ISCIII), INCLIVA, 46010 Valencia, Spain; 3 Cardiology Department, Hospital Universitari i Politècnic La Fe, 46026 Valencia, Spain

**Keywords:** extracellular vesicles, exosomes, aging, hallmarks

## Abstract

The exponential growth in the elderly population and their associated socioeconomic burden have recently brought aging research into the spotlight. To integrate current knowledge and guide potential interventions, nine biochemical pathways are summarized under the term hallmarks of aging. These hallmarks are deeply inter-related and act together to drive the aging process. Altered intercellular communication is particularly relevant since it explains how damage at the cellular level translates into age-related loss of function at the organismal level. As the main effectors of intercellular communication, extracellular vesicles (EVs) might play a key role in the aggravation or mitigation of the hallmarks of aging. This review aims to summarize this role and to provide context for the multiple emerging EV-based gerotherapeutic strategies that are currently under study.

## 1. Extracellular Vesicles in Aging

### 1.1. What Are Extracellular Vesicles?

Extracellular vesicles (EVs) are fundamental mediators of intercellular communication. They are commonly defined as those particles naturally released from a cell, delimited by a lipid bilayer, which do not contain a functional nucleus and therefore lack replicative capacity [1]. EVs contain a wide range of biomolecules, including proteins, lipids, metabolites, and nucleic acids [2]. Compared to soluble mediators, EVs provide their cargo molecules with longer half-lives and protection from degradation by extracellular enzymes. Additionally, they allow their content to be targeted to a specific tissue or organ, as well as their origin to be tracked [3]. These unique traits have recently sparked interest in EVs in virtually every field of biology, from cancer to age-related diseases. Since alterations in intercellular communication are crucial to age-related dysfunction, many authors have investigated how EV-mediated communication might affect and be affected by the aging process [4,5]. 

In the past, EVs have been classified according to their biogenetic pathway into exosomes, microvesicles, and apoptotic bodies [2]. Exosomes are formed by the endocytic budding of the cell membrane, producing an intra-luminal vesicle that is loaded with a particular cargo. Then, in an endosomal complex required for transport (ESCRT)-dependent or -independent pathway, this multivesicular body fuses with the plasma membrane, releasing exosomes into the extracellular space. Microvesicles are formed by the direct outward budding as well as fission of the membrane and are similar to apoptotic bodies for cells undergoing this cell death pathway; however, since defining a biogenetic route can be challenging, a classification based on physical features, including size, has recently been proposed by the International Society for Extracellular Vesicles (ISEV), becoming the most widely accepted one [1]. Although most research involves EVs of 100 nm and above [6], their size can range from as little as 40–50 nm in diameter. According to the ISEV, EVs under 200 nm in diameter should be considered as small EVs, while those over 200 nm are medium or large EVs. Other classifications based on physical properties include density (low, middle, or high) and biochemical composition (for instance, CD63+/CD81+ EVs or annexin-A5-stained EVs). Characteristics of donor cells, such as cell type or culture conditions, are also acceptable as sorting rules (such as podocyte EVs, hypoxic stem cell EVs, or UV-treated fibroblast EVs) [1]. EVs are capable of mediating autocrine, paracrine, and endocrine communication. Target cell specificity is usually determined by specific interactions between proteins enriched at EVs’ surfaces, such as integrin heterodimers, and receptors in the cell membrane or extracellular matrix (ECM), such as ICAMs and fibronectin or laminin, respectively [7]. Once in the recipient cell, EVs can exert their effects in two ways: extracellularly, through interactions with membrane receptors, or intracellularly, via direct cargo release into the cytoplasm after endocytosis [8]. 

EVs’ cargo includes proteins, lipids, nucleic acids, and small-molecule metabolites (amino acids, ATP, NAMPT, and amides) [2,9]. EVs’ protein content is explained to a great extent by its formation process, where the ESCRT plays a key role; therefore, EVs usually contain ESCRT-associated proteins, such as clathrin, ALIX, TSG101, or HSC70 [10]. EVs also inherit standard membrane organizer proteins from parent cells, for instance, tetraspanins CD63, CD9, CD81, TSPAN 6, and flotillin [2]. Cytoplasmic proteins, namely chaperones (Hsp70 and Hsp90), lysosomal membrane proteins (the Lamp2 family), cytoskeletal proteins (actin, myosin, and tubulin), cell-to-cell interaction proteins (integrins, lactadherin, or ICAMs) or enzymes (GAPDH, pyruvate kinase) are usually found in EVs [2,11]. In addition to these structural proteins, EVs contain specific proteins according to their function or cell source, such as APP for neural cells or CXCR4 for immune cells [8]. This has been further investigated by Raposo et al., who showed how antigen-presenting cell-derived EVs are enriched with major histocompatibility complex (MHC-I and II) proteins [12]. 

The lipid bilayer composition in EVs is, to a great extent, shared with that of the plasma membrane, although lipidomic analyses have found EVs to be enriched in glycosphingolipids, sphingomyelins, phosphatidylcholines, phosphatidylserines, and cholesterol in comparison [13,14]. Some of these lipids are highly bioactive molecules involved in intercellular signaling pathways, such as arachidonic acid and its derivates, leukotrienes, and prostaglandins [15]. In terms of genetic material, EVs contain both DNA and RNA in their different forms, including small nuclear RNAs, non-coding RNAs, nucleolar RNAs, ribosomal RNAs, mitochondrial RNAs, and microRNAs, among others [16,17]. The genetic cargo of EVs tends to represent the physiological state of the parent cell. However, the existing variability in both the quantity and type of RNAs in EVs compared to the cell seems to suggest that certain RNAs are selectively incorporated as mediators of intercellular communication [18]. Thomou et al. proved adipose tissue to be a major source of EV-contained miRNAs that regulate gene expression at an organismal level [19]. 

### 1.2. Where Do EVs Come from in Aging Studies?

**Figure 1:**
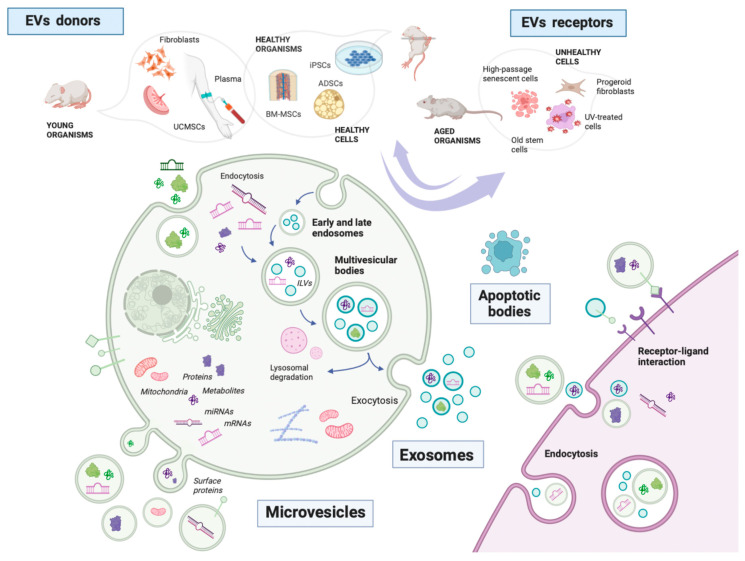
EVs’ origins, receptors, biogenesis, and cargo in aging research. ILVs: intraluminal vesicles; UCMSCs: umbilical cord mesenchymal stem cells; BM-MSCs: bone marrow mesenchymal stem cells; iPSCs: induced pluripotent stem cells; and ADSCs: adipose-derived stem cells. Created with Biorender.com; accessed 12 December 2022.

EVs used in aging research can be classified, according to their origin, as (i) natural or (ii) artificial/modified. Natural EVs are isolated from young, healthy individuals or centenarians, both from body fluids or in vitro cultured cells [20,21]. Donor serum is the most widely used source of EVs, as it allows for the study of systemically distributed metabolites and biomarkers that change in a time-dependent manner [22,23]. Other biological fluids, such as cerebrospinal fluid (CSF) [24,25], saliva [26], or breast milk [27], are currently under study, with the advantage of providing information about the biological pathways that occur with aging in a specific tissue and explaining their therapeutic properties. Cell cultures most often involve stem cells (SCs), although EVs from terminally differentiated cells such as fibroblasts have received some attention too, with donor ages ranging from young [28,29] to centenarian [30]. Of all types of SCs, mesenchymal SCs (MSCs) are preferred, since they are ubiquitous in organisms, they expand rapidly in adherent cultures, and cost-effective isolation methods exist. MSCs used for EV isolation are bone marrow MSCs (BM-MSCs), adipose-derived MSCs (ADSCs), dental pulp MSCs (DP-MSCs), and umbilical cord MSCs (uc-MSCs) [31]. EVs are isolated from culture media of MSCs; culture conditions for optimal MSC growth and purified EV isolation include a low (3% or similar) oxygen concentration [32,33] and the use of exosome-depleted serum [34]. Rarer sources include embryonic SCs (ESCs) [35], amniotic fluid MSCs [36], or neural SCs (NSCs) [37] (Figure 1). Although most studies focus on EVs of mammalian origin, animal non-mammalian EVs, such as those from C. elegans [38], and plant EVs [39] deserve a mention due to the growing evidence of their interest in aging studies. 

EVs from artificial sources are obtained from induced pluripotent SCs (iPSCs) [40,41] or donor cells subject to media conditioning or content modifications. These sources overcome some of the shortages of natural EVs, such as slow production, short half-lives, fast clearance, and weak targeting [4]. iPSCs guarantee a rapidly expanding culture and avoid ethical issues concerning other types of pluripotent cells, such as human ESCs [42]. iPSC-derived EVs have recently been used to regenerate aged skin [43] and ischemic hearts [44] in in vitro and in vivo models, with encouraging results. Media preconditioning alters EVs’ content, which is of great use to either modify or help identify the active components responsible for observed effects. For example, the osteogenic differentiation of MSCs by culturing in a supplemented medium can help produce osteogenic EVs capable of promoting bone regeneration [45]. The coating improves the passage of EVs through physiological barriers, as shown by coated EVs with improved skin penetration for photoaging treatment [46], and improves EVs’ specificity towards a certain tissue, such as bone, with aptamer-coated EVs [47].

As for delivery routes, the most frequently used are intravenous and intraperitoneal [48]. Less invasive routes, such as oral or topical, have also been proven to be safe and effective since EVs escape the usual degradation mechanisms in the digestive tract [49]. More complex routes are EV-containing hydrogel injection or bioactive scaffold embedding, for longer half-lives in vivo [50]. Regarding EV isolation methods, they can be based on density, size, immunoaffinity, solubility, or aggregation properties. While serial ultracentrifugation is generally considered the gold standard, alternative methods, such as ultrafiltration, size exclusion chromatography, and immunoprecipitation, have been proven to be efficient, precise, and less time-consuming, at a usually higher cost [32,51]. 

## 2. EVs and the Hallmarks of Aging

### 2.1. What Are the Hallmarks of Aging?

The term hallmarks of aging was coined by López-Otín et al. in 2013 to define the biochemical changes that occur in all organisms that experience biological aging and which lead to a progressive loss of physiological integrity, impaired function, and, eventually, death [52]. To be termed a hallmark, a biochemical process must fulfill three characteristics: (i) it should be found during physiological aging, (ii) its experimental exacerbation should accelerate aging, and (iii) its experimental downregulation should mitigate it, hence increasing the healthspan and even lifespan [52]. This last criterion poses a real challenge to the definition; in fact, few of the hallmarks are yet backed up by generally accepted interventions. The importance of hallmarks is double: first, they set a framework for aging studies, putting a focus on the most relevant molecular processes; second, they provide research with molecular targets for aging interventions. Traditionally, nine processes are accepted to meet these criteria: genomic instability, telomere attrition, epigenetic alterations, loss of proteostasis, deregulated nutrient sensing, mitochondrial dysfunction, cellular senescence, stem cell exhaustion, and altered intercellular communication. Recently, some more processes have been proposed to join this list, such as in the cases of disabled macroautophagy, chronic inflammation, dysbiosis [53], the dysregulation of RNA processing, and altered mechanical properties [54]. The role that EVs naturally play in those hallmarks, as well as their potential to modulate them, is the subject of this review. 

### 2.2. Primary Hallmarks

The primary hallmarks could be the initiating triggers whose damaging consequences progressively accumulate with time. The common characteristic of primary hallmarks is that when they appear they are unequivocally negative. This is the case with DNA damage, telomere loss, epigenetic drift, and defective proteostasis [49].

#### 2.2.1. EVs and Genomic Instability

A common feature of organismal aging is the accumulation of genetic damage [55]. Throughout life, DNA integrity and stability are continuously compromised by exogenous (physical, chemical, and biological agents) and endogenous factors, such as errors in the replication process or the threat of reactive oxygen species (ROS). The genetic lesions caused by this damage are very diverse, namely point mutations, translocations, chromosomal gains and losses, and telomere shortening. Organisms have therefore developed several DNA repair mechanisms in order to minimize these lesions [56,57]. However, these lesions accumulate over time and cell function is finally affected, thus altering tissues’ homeostasis [52]. EVs may play an important role in inducing genomic instability in aging-related diseases, such as cancer, and thus be important factors in the pathogenesis of these diseases [58]. EVs released by leukemia cells can cause DNA damage and the instability of normal cells via the transfer of BCR-ABL1 mRNA, leading to genetic alterations and ultimately malignant transformations. Tumor EVs have also been shown to contain oncogenic DNA, which is transferred to target cells, where abnormal foci of micronuclei are formed [59,60]. 

**Figure 2:**
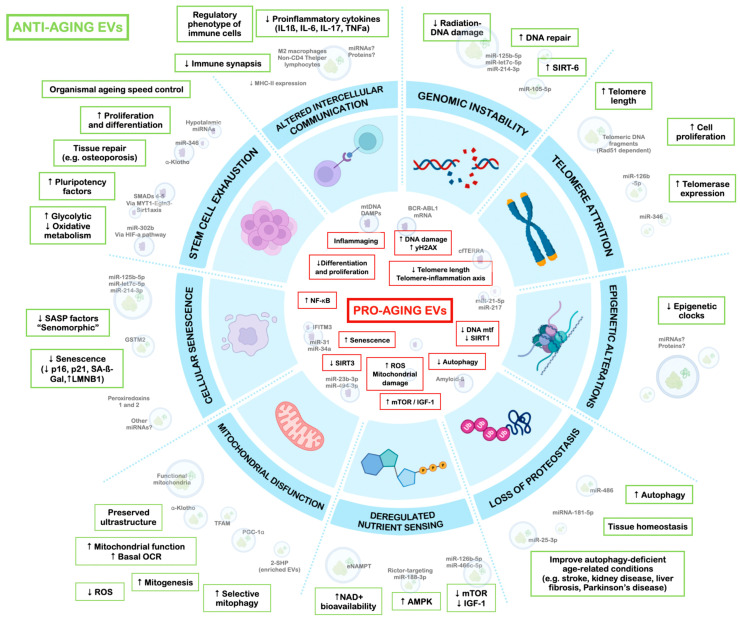
Graphical summary of EVs’ pro-aging (red boxes) and anti-aging (green boxes) effects on the different hallmarks, in addition to the active cargo responsible for the observed effects. Based on the original figure by Lopez-Otín et al. [52]. Created with BioRender.com.

On the other hand, several studies have observed an improvement in markers of DNA integrity and stability after treatment with EVs derived from MSCs in different models where genetic material was subjected to damage. Zhu et al. observed a decrease in DNA damage marker proteins such as 8-hydroxy-deoxy deoxyguanosine (8-OHDG) and histone γH2AX in intestinal tissue after the intravenous administration of EVs derived from rat bone mesenchymal stem cells (BMSCs) in rats with induced ulcerative colitis [61]. Similarly, MSC-EVs decreased these same markers’ levels in different in vitro models, such as kidney epithelial cells or microglial cells after radiation damage [62,63,64]. Recently, Sun et al. have shown that iPSC-EVs promote DNA repair through the activation of sirtuin 6 (SIRT6) in an in vitro model of nucleus pulposus cells [65]. In summary, EVs secreted by MSCs of different origins appear to promote DNA repair after different injuries (Figure 2).

#### 2.2.2. EVs and Telomere Attrition

In cases of damage, certain chromosomal regions are more susceptible than others to trigger aging-related pathways. Telomeres are the most obvious example since they lack repair capacity by standard DNA polymerases; instead, they require telomerase, an enzyme generally missing in adult cells, to regenerate [66]. The shortening of telomeres with physiological aging in humans [67], its relationship with several diseases, such as pulmonary fibrosis or aplastic anemia [68], and its interest as a mortality risk predictor [69] have been widely studied. In animal models, genetic manipulations that shorten or lengthen telomeres result, respectively, in decreased [70] or increased longevity [71]. The strongest evidence for the critical role of telomeres in aging was provided in 2012 when Bernardes de Jesus et al. reported that telomerase gene therapy could increase the healthspan and lifespan of mice without increasing the incidence of cancer [72]. Telomere attrition can actively drive most hallmarks, from mitochondrial function to senescence and stem cell integrity [73], to the extent that some authors agree with a telomere-based rationale in the hallmarks of aging [74]. Several well-known aging interventions, namely dietary restrictions, exercise, and rapamycin, have been shown to improve telomere status by reducing the number of telomere-associated damage response foci (TAFs) [75]. According to the most widespread hypothesis, telomere shortening does not lead to aging or age-related diseases per se; instead, damage to telomeric DNA activates cellular senescence pathways, causing age-related tissue damage that is further magnified through their senescence-associated secretory phenotype (SASP) to adjacent cells or even distant tissues [74]. For this reason, all elements implied in cell-to-cell communication are increasingly appealing. 

In particular, EVs’ involvement in telomere shortening has just recently attracted some attention. Some studies have investigated a type of non-coding RNA, telomeric-repeat-containing RNA (TERRA), that is overexpressed in the cytoplasm as telomere length decreases, due to increased TRF2 [76]. In these works, cell-free TERRA (cfTERRA)-loaded EVs are proven to boost the innate immune response and pro-inflammatory cytokine expression in peripheral blood cells [77,78], hence establishing a link between telomeres and inflammation. In another study, EVs derived from radiated cells were proven to reversibly reduce telomerase activity in recipient cells, mediating a bystander effect; the specific mechanism remains unclear, but proteins and miRNA cargo are suggested to play a role since treatments with both RNases and protein denaturalization through boiling were able to impair these results [79].

On the other hand, EVs may be able to intervene in the telomere attrition process in target cells under certain conditions. In two different works, Sonoda et al. treated ovariectomized mice [80] and mice with systemic lupus-erythematosus-like disease [81] with stem cells from human exfoliated deciduous teeth (SHED) and their EVs. SHED-EVs mimicked the effects of a SHED transplant on bone marrow mesenchymal stem cells (BMMSCs) of mice: they were able to rescue *Tert* mRNA expression and telomerase activity, improving their function and stemness. Moreover, ribonuclease-treated EVs do not achieve similar results, thereby pointing to a particular miRNA (miR-346) as being responsible for these effects. Another in vivo study on old mice injected with EVs from the serum of young mice showed an upregulation of telomerase-complex-related genes (*Men1* and *Mre11a*) in the liver and lungs [82]. The authors postulate miRNAs, particularly mmu-miR-126b-5p, as the active components behind these effects. Nonetheless, a measure of telomere length is missing in both works to support these results. In more recent work, aged mice were treated with exosomes from young ADSCs; the authors found no differences in either mean telomere length or telomere damage [83]. In vitro studies yield more promising results. In a major work in the field, Lanna and colleagues revealed that some T cells elongate their telomeres thanks to an EV-mediated donation of telomere fragments from antigen-presenting cells [84]. This elongation did not depend on telomerase action; instead, the Rad51 combination factor mediated the fusion of base pairs to chromosomal ends. As a consequence of this transfer, the population of senescent T cells decreased in vivo, parallel to an increase in T cell proliferation rates and a stem-like T cell memory switch. Longer immunological memory and better survival in these mice in response to infection resulted from T cell telomere elongation [84]. In addition to this telomerase-independent pathway, a telomerase-dependent pathway of telomere repair has been discovered in a cancer model. Researchers showed how cancer cells can package an mRNA transcript of human telomerase reverse transcriptase (hTERT) in EVs and release it to the extracellular medium; hTERT is then translated into fully active telomerase in normal fibroblasts and prompts their conversion into cancer-associated fibroblasts via microRNA transcriptome reprogramming [85] (Figure 2). 

Thus, the role of EVs in telomere attrition should be considered in the field of aging and age-related diseases. 

#### 2.2.3. EVs and Epigenetic Alterations

Epigenetic alterations, such as histone acetylation and methylation or DNA methylation pattern changes, constitute specific age-associated epigenetic marks [52,86]. EVs can induce epigenetic changes in recipient cells, as is the case of tumor-derived EVs, which are capable of modulating the tumor microenvironment and inducing the transformation of normal cells [87]. Zhu et al. observed that EVs derived from tumor cells induced the hypermethylation of promoters of tumor suppressor genes P53 and RIZ1 in healthy cells [60]. In another study with replicative senescent human umbilical vein endothelial cells (HUVECs), EV-contained miR-21-5p and miR-217 downregulated the expression of DNA methyltransferase 1 and SIRT1, two enzymes that cooperate to maintain genetic methylation patterns, in young cells, resulting in high levels of SASP molecules and cell cycle inhibitors [88]. On the other hand, EVs may also exert an anti-aging effect. Our group has recently observed that the treatment of EVs from adipose-derived stem cells (ADSCs) from young mice rejuvenates the epigenetic clock of liver and kidney tissues from old mice by changing the DNA methylation pattern [83]. To our knowledge, this study is the first to show that EV intervention can decrease epigenetic age in an old organism (Figure 2).

#### 2.2.4. EVs and Loss of Proteostasis

Cells have mechanisms that regulate protein homeostasis by maintaining a stable and functional proteome. Under stress conditions, surveillance systems allow for detecting and repairing damaged proteins or, in some cases, their elimination [89]. One of the most important mechanisms for maintaining proteostasis is autophagy, which is responsible for the removal of abnormal proteins. It is known that autophagic activity decreases with age, contributing to the accumulation of damaged proteins and organelles during aging. This can lead to the development of age-related diseases, such as Alzheimer’s disease (AD) [90,91]. Here, EVs secreted in CNS may play an important role in pathogenesis, favoring the propagation of toxic amyloid proteins and thus inducing a loss of proteostasis [92,93]. EVs have also been found to be responsible for aging-related autophagy disruption in non-mammalian models. According to this study, mir-83, which is responsible for the regulation of macroautophagy across tissues in C. elegans, is upregulated in the intestine with aging and impairs autophagy through the suppression of CUP-5/MCOLN [94].

Autophagy is related to the synthesis of EVs as some autophagy-related proteins, such as ATG5, are also involved in the biogenesis of extracellular vesicles [95]. The role of mammalian MSC-EVs in autophagy is controversial. For instance, MSC-EVs have been shown to increase autophagy in models of age-related diseases, such as diabetic nephropathy, liver fibrosis, cardiac ischemia, and Parkinson’s disease (PD) [96,97,98,99,100]. However, Kuang. et al. observed that ADSC-EVs reduced autophagic flux in an in vitro model of stroke [101]. These studies seem to indicate that the role of MSC-derived EVs in autophagy may depend on several factors (tissue, types of MSCs, previous damage, etc.), although further studies are needed (Figure 2). 

### 2.3. Antagonistic Hallmarks

In contrast to the primary hallmarks, antagonistic hallmarks have opposite effects depending on their intensities. At first, at low levels, the antagonistic hallmarks are initially beneficial but become progressively negative in a process that is in part promoted or accelerated by the primary seals that mediate the beneficial effects. For instance, senescence protects an organism from cancer, but over time its excess can promote tissue aging. Similarly, ROS can mediate cell signaling and survival but produce cellular damage at chronically high levels; likewise, optimal nutrient sensing and anabolism are important for survival but, in excess and over time, can become pathological [49].

#### 2.3.1. EVs and Deregulated Nutrient Sensing

Some of the best-preserved evolutionary mechanisms of longevity control are the nutrient sensing and growth-related pathways, such as the insulin/IGF-1, mTOR, AMPK, and sirtuin pathways [52,102,103]. Hence, interventions that affect these pathways, such as dietary restrictions, mTOR inhibition through rapamycin, or AMPK activation through metformin, have beneficial effects on longevity [104,105,106]. EVs have been postulated as mediators of these pathways in some studies. Lee et al. observed that plasmatic EVs from young mice reduce mTOR and IFG1 receptor levels in the liver and lungs of old mice [82]. Similarly, EVs derived from MSCs can also reduce mTOR levels and induce AMPK activation, thereby increasing autophagy [99,107]. By contrast, the incubation of young MSCs with old MSC-EVs has been shown to activate mTOR pathways, suggesting that the mechanism is bidirectional [28]. Fafián-Labora et al. obtained similar results with young and old MSC-EVs; interestingly, they proposed miR-188-3p, which targets the mTOR-related factor Rictor, as being responsible for the mTOR inhibition, AMPK promotion, and improved pluripotency of these cells [108]. 

It has also been described that the bioavailability of some metabolites, such as NAD^+^, declines with age, reinforcing DNA damage and inflammation pathways [109]. In a key study in the field, Yoshida et al. observed that extracellular nicotinamide phosphoribosyltransferase (eNAMPT), a nicotinamide adenine dinucleotide (NAD^+^) biosynthetic enzyme, was found in EVs, but its concentration dropped over time [22]. Previous studies have shown that eNAMPT secreted by adipose tissue, promoted by situations such as dietary restrictions in a SIRT1-dependent pathway, could increase NAD^+^ in the hypothalamus, improving physical tests in mice [110]. In their work, Yoshida et al. treated old mice with eNAMPT-containing EVs from young mice plasma and observed significant improvements in lifespan and healthspan. These studies suggest that EVs from both young mice and those derived from MSCs have effects on aging-related metabolic pathways, promoting longevity [22] (Figure 2).

Accordingly, several studies have shown that EVs may contribute to the appropriate balance of energy and nutrient sensing. 

#### 2.3.2. EVs and Mitochondrial Dysfunction

As cells age, mitochondrial efficiency decays, which is measurable as a decrease in ATP production and an increase in electron leakage [111]. Conversely, mitochondrial dysfunction has been shown to drive premature aging in mammals [112]. This bioenergetic impairment is potentially toxic for the cell through ROS production, pro-inflammatory signaling, or mitochondrial membrane permeabilization, leading to DNA damage or apoptosis pathways. The loss of mitochondrial function with aging results from the combination of impaired biogenesis and reduced turnover [111]. Mitochondrial biogenesis is tightly linked to telomere damage and sirtuin regulation, as it frequently occurs in response to the p53-mediated repression of PGC-1α [113]. The accumulation of damaged mitochondria is generally prevented by mitophagy, a type of organelle-specific macroautophagy that appears to be impaired in some tissues in aging and age-related diseases. Interventions that promote mitophagy, such as caloric restriction [114] or compounds similar to tomatidine [115], constitute a promising anti-aging approach [116]. 

Mitochondrial-derived vesicles (MDVs) are emerging actors in the mitochondrial quality control system. While mitophagy is mainly triggered by severe mitochondrial damage, MDVs allow for the elimination of components in mildly damaged organelles or support mitophagy when the process is overwhelmed or compromised [117]. Hence, mitochondrial quality control depends on an equilibrium between extracellular mitochondrial release and mitophagy [118]; failure in this system may lead to inflammaging, as shown in studies where PD patients showed lower levels of MDVs than healthy controls [119]. On the contrary, other studies suggest that mitovesicles themselves may be to blame for this inflammatory response [120]. It has been suggested that when mitochondrial DNA (mtDNA) is freely released from the cell, it acts as a ligand of NRL3 and triggers inflammation cascades [117]. Aging studies show that EV-contained mtDNA declines with aging, inversely proportional to cell-free mtDNA, and this may contribute to chronic inflammation [121]. Furthermore, it has been proposed that EVs of certain origins may be able to impair energetic networks in the receptor cell. In a recent study, HeLa cells treated with EVs from young and old individuals showed an improved or worsened oxygen consumption rate, respectively [121]. Similarly, in a model of idiopathic pulmonary fibrosis (IPF), lung-fibroblast-derived EVs were able to increase mitochondrial ROS and cause mitochondrial damage via miR-23b-3p and miR-494-3p, which suppress SIRT3 expression [122]. 

EVs have also been postulated to enhance the mitochondrial function of recipient cells. Sahu et al. observed that aged muscle-derived progenitor cells (MDPSCs) cultured with young serum EVs increased their basal oxygen consumption rate when compared to cells exposed to aged serum; furthermore, mitochondrial ultrastructure was also improved, as demonstrated by an increase in the reduced form of the mitochondrial membrane phospholipid cardiolipin [123]. Another work with MDPSC-EVs proved that they are capable of restoring mitochondrial function following oxidative injury, while not affecting the function of healthy myotubes [124]. 

In a notable work focusing on neural tissue, the authors found that EVs mediate the transfer of functional mitochondria between NSCs, explaining the benefits of NSC transplantation in some neurodegenerative diseases. In their model, EVs rescued the mitochondrial function of target cells, increasing survival; moreover, these healthy mitochondria were taken up by immune cells, reducing the expression of pro-inflammatory markers [125]. Similarly, MSC-EV-mediated mitochondrial transfer has also been proposed as a repair mechanism in acute respiratory distress syndrome (ARDS) models of lung injury [126]. Congruent results were observed in a model of myocardial infarction, where EVs from autologous-stem-cell-derived cardiomyocytes restored ATP production and improved contractile profiles of hypoxia-injured cardiomyocytes; while mitochondrial cargo might play a role, the authors also propose mitochondrial-biogenesis-related messengers, such as PGC-1α, as potential actors [127]. In an in vivo model of kidney injury, MSC-EVs rescued mitochondrial damage via the transfer of mitochondrial transcription factor A (TFAM) [128]. As for the role of engineered EVs, recent work has obtained promising results with protein tyrosine phosphatase 2 (SHP2)-enriched MSC-EVs in a model of AD by selectively inducing mitophagy in neural cells. As a result, neuronal cell apoptosis and inflammation are decreased, which culminates in lower synaptic loss and cognitive decline [129] (Figure 2). 

Therefore, mitochondrial-derived vesicles in aging and age-related diseases are very important as markers, but also as possible mediators for specific treatments. 

#### 2.3.3. EVs and Cellular Senescence

Cellular senescence is defined as a permanent state of cell cycle arrest that occurs in response to different noxious stimuli, namely telomere attrition, DNA damage, certain oncogene expression, altered chromatin organization, and strong mitogenic signals [130]. As a result, cells are endowed with a specific phenotype, consisting of an enlarged irregular morphology, loss of nuclear membrane protein lamin B1, DN-damage-associated γH2AX foci, lysosome accumulation with increased senescence-associated ß-galactosidase (SA-ß-Gal) activity, the overexpression of the cell cycle inhibitors p16Ink4a and p21, and the phosphorylation of p53 [131]. In addition, they acquire a specific senescence-associated secretory phenotype (SASP), with the enriched secretion of inflammatory cytokines, that contributes to the spread of senescence to adjacent cells in a paracrine manner [132]. 

The accumulation of senescent cells negatively affects the regenerative capacity of tissues and creates a pro-inflammatory microenvironment favorable to the progression of age-associated conditions, such as fibrosis or cancer [133]. Nonetheless, senescent cells are not exclusive to aging organisms; in fact, senescence is present from the earliest stages of life and plays a fundamental role in tissue homeostasis by promoting organ remodeling and protecting against tumorigenesis. This positive role is arguably lost during aging, as immune dysfunction and stem cell exhaustion hinder the removal of senescent cells [134]. The well-known accumulation of senescent cells in most tissues [135] has placed them at center stage among potential anti-aging aging targets. Two approaches have emerged to this end: senolytics, which seek to eliminate senescent cells by promoting apoptosis or immune clearance, and senomorphics, which attempt to limit paracrine effects through SASP modulation [136]. Studies with the senolytic transgene INK-ATTAC, which can trigger the clearance of p16(Ink4a)-positive cells, revealed improved healthspans and lifespans in mice [137], as did the senolytic drugs dasatinib–quercetin [138]. Senomorphic compounds, such as metformin, have also repeatedly been shown to improve healthspans and lifespans in long-term treatments in mice [139]. 

As for the role of EVs, cellular senescence is probably the hallmark that has attracted the most attention to date. Even though the best-known SASP components are soluble factors, such as pro-inflammatory interleukins (IL-1α and ß, IL-6, and IL-8), growth factors (FGF, TGF-ß), chemokines (CCL-2, CXCL-2, and TNF-α,) or extracellular remodelers (MMP-1, VEGF), EVs are also crucial actors of SASP (defining the so-called evSASP) [140,141]. It has long been known that EV secretion increases up to 50-fold in senescent cells [142], presumably due to p53 activation [143]. This enhanced secretion could be indispensable to maintaining cellular homeostasis by excreting harmful DNA and preventing apoptosis-like cell death [144]. EVs are potential players in the so-called bystander effect, where damaged cells seem to transmit senescence to adjacent ones [145]. Some attribute this effect to genetic material, while others set the focus on proteins. In a recent study, miR-31, an overexpressed miRNA in EVs from senescent endothelial cells, was discovered to inhibit the osteogenic differentiation of MSCs [146]; similarly, senescent muscle-cell-derived EVs, both in vitro and in vivo, were found to overexpress miR-34a, which induced senescence and decreased the SIRT1 expression of BMSCs [147]. Borghesan and colleagues found that IFN-induced transmembrane protein 3 (IFITM3), which is selectively packed in EVs from senescent cells, could induce paracrine senescence in healthy cells [148]. Downstream signaling pathways activated by evSASP remain largely unknown, but some authors highlight the contributions of NF-κB/IKK factors [149]. The capacity of EVs to activate NF-κB-derived pathways has also received attention in models of non-fatty liver disease and atherosclerosis [150]. Evs of rarer origin, such as ginseng-root-derived EVs, have also shown senotherapeutic effects on human fibroblasts and melanocytes, where senescence was induced by high replication rates or UV radiation, respectively [39].

Nevertheless, the role of EVs as SASP factors is largely offset by their enormous potential as senotherapeutics. In one of the most remarkable in vitro studies, Fafián-Labora et al. observed that EVs from young cells ameliorated senescence-related biomarkers in fibroblasts from old or Hutchinson–Gilford progeria donors. Similar effects were investigated in tissues, such as liver, kidney, lung, or brown adipose tissue, in a mice model. They attribute these effects to an antioxidant enzyme, glutathione-S-transferase mu 2 (GSTM2), mainly found in small EVs, whose activity tends to decrease as an organism ages. Together with the senescence markers p16, p21, SA-ß-Gal, and yH2AX, reactive oxygen species (ROS) levels and lipid peroxidation markers were decreased after treatment [28]. This antioxidant theory is supported by one study that found EVs decreased the senescent phenotype of MSCs thanks to the delivery of peroxiredoxins 1 and 2 [151]. Moreover, EVs obtained from plants with well-known regenerative properties, such as aloe vera, were also shown to increase antioxidant activity, measured through superoxide dismutase (SOD) and ROS in human dermal fibroblasts via Nrf2 activation [152]. Congruent results have been obtained with EVs from stem cells. In recent work, researchers found that EVs from young MSCs also decreased most senescence markers in tissues from aged mice. Even though they do not propose a single EV-contained mediator, they propose that EVs act as senomorphics by downregulating the expression of SASP factors [153]. Some experiments in UV-radiated fibroblasts also suggest the senotherapeutic role of iPSCs or MSC-EVs in skin aging, although the underlying mechanism remains elusive [40,154]. Interestingly, Sanz-Ros et al. made similar findings in in vivo and in vitro models. The treatment of old mice with young ADSC-sEVs reduced levels of the senescence markers lamin B1 and γH2AX in kidney and muscle, as well as protein and lipid oxidation. SASP factors were also decreased in these tissues; hence, the authors suggest a senomorphic effect of EVs. EV-contained miRNAs were hypothesized to mediate these results, since several differentially expressed miRNAs in young EVs proved capable of reducing senescence biomarkers in a myoblast culture. Two of the identified miRNAs, miR-214-3p and miR-125b-5p, have already shown beneficial effects on fibroblast function during wound healing and angiogenesis in endothelial cells [29,155] (Figure 2). 

### 2.4. Integrative Hallmarks

The integrative hallmarks comprise stem cell exhaustion and altered intercellular communication, which affect tissue homeostasis and function. These hallmarks arise when the accumulated damage caused by the primary and antagonistic hallmarks cannot be compensated by tissue homeostatic mechanisms [49].

#### 2.4.1. EVs and Stem Cell Exhaustion

The age-related decline in tissue regenerative capacity is largely due to the functional attrition of underlying stem cells [156]. SCs of different origins, such as HSCs [157], NSCs [158], or MDSPCs [159], have shown a decrease in cell cycle activity and differentiation courses with time. This exhaustion arises from both intrinsic, namely DNA damage or telomere shortening, or extrinsic stimuli, as is the case of alterations in the cell niche or circulating factors [160]. Evidence is growing for the crucial role of the latter [161], especially since the first parabiosis experiments showed that the function of aged neural or muscle SCs could be recovered through exposure to the systemic factors of young mice [161,162]. In line with these works, researchers found that the transplantation of MDSPCs from young mice could extend the lifespans of mice with progeria. Interestingly, degenerative changes were reversed in tissues where transplanted cells did not engraft, particularly muscle, suggesting that their therapeutic benefit may derive from secreted factors [163].

The question is therefore raised as to whether EVs hold a place among these factors. Recent work comparing young BM-MSC transplantation and young MSC-EV treatment report similar results; MSC-EVs can account for the observed effects of MSCs on lifespan extension and rescue old MSCs’ function in mice. Moreover, researchers observed that EVs from young MDSPCs could also decrease senescence in MDSPCs from progeroid mice, consequently improving differentiation potential and myotube formation [153]. In another in vivo study, human ESC-EVs were able to rescue bone loss by promoting the proliferation and osteogenic differentiation of BM-MSCs in old mice; the authors proposed that EV-contained proteins synergistically modulate the expression of anti-aging genes in BM-MSCs [35]. Evidence is still more relevant as far as neural tissue is concerned, since studies have revealed that aging speed in mammals is regulated by hypothalamus control through EV-contained miRNAs [37]. In vivo studies have shown that hypothalamic NSCs (hNSCs) suffer depletion with aging; accordingly, young NSC transplantation extended their lifespan in mice. Treatment with NSC-EVs mimicked the effects of SCs, improving healthspan parameters such as resistance to fatigue, motor coordination, memory, and sociality tests [37]. Further investigations explain this effect as a result of the decreased senescence of hNSCs [164]. Some authors have demonstrated that ESC-EVs could rescue hNSCs’ stemness by transferring SMADs, which activate HIF-2α, NAMPT, and Sirt1 successively via myelin transcription factor 1 (MTF1), downregulated in aged hypothalamic NSCs [165]. Sanz-Ros et al. also found increased proliferation in kidney tubules after systemic treatment with ADSC-EVs [83]. Muscle function is of great interest due to its tight relation to frailty, one of the strongest mortality predictors in the elderly [166]. In their work, Sanz Ros et al. found that muscle cross-sectional area and protein content were increased upon ADSC-EVs treatment; this translated into improved physical tests and decreased frailty scores [83].

As for in vitro experiments, Sahu et al. studied the effects of young serum EVs on old MDSPCs and observed a marked increase in myogenic differentiation potential after treatment; they attributed the effects to EV-contained α-Klotho transcripts, which decrease in plasma in an age-dependent manner and are key to muscle regeneration [123]. Mas-Bargues et al. found that in vitro treatment with DP-MSC-EVs helped high-passage or hyperoxia-induced senescent MSCs recover their stemness, as evidenced by pluripotency factor (OCT4, SOX2, KLF4, and cMYC) overexpression. They also observed a shift to a more glycolytic and less oxidative metabolism, which is typical of MSCs [33] (Figure 2). 

#### 2.4.2. EVs and Altered Intercellular Communication

During aging, intercellular communication changes at all levels: autocrine, paracrine, endocrine, and neuroendocrine. This deregulation tends to result in increased inflammatory reactions, decreased immunosurveillance against pathogens and premalignant cells, changing compositions of the pericellular and extracellular environments, and therefore an alteration in tissue homeostasis [52,167]. A prominent aging-associated alteration in intercellular communication is the development of a chronic and systemic inflammation process known as inflammaging. This inflammaging situation contributes to the development of age-related diseases such as obesity, type 2 diabetes, atherosclerosis, and AD [168]. Regarding the role of EVs in age-associated chronic inflammation, EVs derived from senescent cells are important elements of the SASP; as such, they spread senescence and contribute to the development of chronic inflammation associated with age [149]. 

However, EVs from healthy and young cells have been shown to exert a protective effect against inflammaging. Tsukamoto et al. found that miR-129 in serum circulating EVs, which is overexpressed in aged EVs, compensates for the hyperinflammatory state associated with aging through a negative feedback loop via NfkB inhibition [169]. Different studies have shown the beneficial effect of MSC-EVs in models of physiological aging [83,153] or models of aging-associated diseases, such as myocardial infarction [170], osteoarthritis [171], and ulcerative colitis [61]; these studies all reveal a decrease in pro-inflammatory cytokines levels, such as IL-6, IL-1, TNF-α, and PGE_2_. Wang et al. also observed that EVs from the serum of young mice were able to decrease inflammaging in old mice by reducing serum levels of IL-6, IL-1, and TNF-α and by lowering to normal levels type-1 T-helper phenotype (CD4+IFN-γ+ T cells). Mice treated with EVs also showed decreased levels of CNS-penetrating CD3+ T cells and macrophages in addition to MHC-II expression by microglia. In addition, the predisposition to autoimmune chronic diseases was reduced by decreasing the concentration of serum anti-nuclear antibodies and inflammatory cell infiltration in the salivary glands [23]. It has been proposed that the protective effect against inflammation exerted by these EVs is due to the inhibition of SASP, acting as senomorphic agents, thus reducing senescence and age-associated inflammation [98,153]. 

Damaged intercellular communication not only affects an individual’s own cells but also its microbiome. The gut microbiome has recently been attributed a key role in the maintenance of organismal homeostasis through immune regulation and endocrine signaling [172]. The change in the composition of the microbiome that occurs with aging has been associated with a number of pathologies, such as obesity, type 2 diabetes, cardiovascular disease, cancer, and neurological disease [173]. Indeed, microbiota transplantation has been shown to increase longevity in HGPS mice [174]. Recent studies show that some of the protective effects of the microbiome on aging may be EV-mediated: EVs from Akkermansia muciniphila (Akk) are responsible for the positive effects of young gut microbiota on bone mass, since released EVs augment osteogenic activity and inhibit osteoclast formation in ovariectomized mice [175,176]. Moreover, their metabolic effects might also be EV-mediated, as shown by a work where Pseudomonas panacis-derived EVs were closely associated with metabolic disease in mice, while Akk-EVs rescued diet-induced obesity and diabetes, both well-known age-related diseases [176].

Table 1 below summarizes the current evidence on the potential roles and effects of EVs on the different hallmarks of aging, according to experimental models (in vitro or in vivo, EVs’ origins, and receptor cells or organisms), the observed changes at the cellular and organismal levels, and the proposed EV components responsible for these effects (Figure 2).

## 3. Conclusions and Perspectives

The current work reviews the hallmarks of aging through the lens of extracellular vesicle biology and reveals how altered intercellular communication underlies each of the hallmarks of aging. Undeniably, EVs are key players in the generation and magnification of tissue damage, which ultimately lead to organismal aging. Pro-aging EV content would promote DNA damage, upregulate mTOR and inhibit AKT pathways, spread pro-inflammatory genetic material, encapsulate SASP factors, and impair stem cell function. Nonetheless, EVs have also increasingly demonstrated the potential to target each of the hallmarks of aging. This is the case for anti-aging EVs, whose content enhances DNA repair, recovers telomeres, promotes autophagy, behaves senomorphically, improves mitochondrial bioenergetics, and rescues stemness. Therefore, EVs have a double-sided role in the aging process. In vivo, the circulating pool of EVs might represent a combination *of pro-aging* and anti-aging EVs in a spectrum that reflects the physiological or pathological statuses of different tissues. As time-dependent cell damage increases, this balance could be disrupted in favor of pro-aging EVs, which are positive feedback to age-related dysfunction [5]. Therefore, EV-based gerotherapeutic strategies have a double use: while some may seek to tackle pro-aging EVs or EVs’ components, others would aim to introduce anti-aging ones from healthier donors. Either way, further research is required to identify those components that account for the observed effects. A solid framework should then be established to differentiate EVs according to their aggravating or mitigating effect on the aging process, to fully unlock their gerotherapeutic potential.

## Figures and Tables

**Table 1:** Potential roles and effects of EVs on the hallmarks of aging. Pro-: pro-aging; anti-: anti-aging; IVT: in vitro; and IVV: in vivo.

*Hallmark*		*Pro-/Anti-*	*IVV/IVT*	*EVs’ Receptor*	*EVs’ Donor*	*EV Cargo*	*Cell-Level and* *Organismal-Level Effects*	*Ref.*
***P*** ***R*** ***I*** ***M*** ***A*** ***R*** ***Y***	**Genomic ** **instability**	Pro-	IVT	Human mononuclear cells from healthy donors	K-562 cell line (lymphoblasts)	NA	Inhibited tumor suppressor genes P53 and RIZ1. Activated cytidine deaminase and ROS, leading to DNA breakage and recombination Treated cells exhibit a leukemia-like malignant phenotype	[55]
			IVT	HUVECs	RAS-3 cell line (intestinal epithelial cell line containing c-HRAS oncogene)	BCR-ABL1 mRNA	Increased the level of DNA damage response markers, such as the phosphorylation of histone γH2AX Increased oncogenic transformation potential	[54]
		Anti-	IVV	Intestinal epithelial cells	BM-MSCs	NA	Limited intestinal epithelial cells’ ROS accumulation and DNA damage Alleviated ulcerative colitis injury	[56]
			IVT	FDC-P1 cell line (bone marrow hematopoietic cells)	BM-MSCs	miR106b-3, miR155-5p, and miR210-5p	Rescued radiation-associated DNA damage	[57]
			IVT	Renal epithelial cells	h-UCMSCs (human umbilical cord MSCs)	NA	Decreased DNA damage foci and senescence	[52]
			IVT	Microglia from adult rats (primary culture)	ADSCs	NA	Activation of SIRT1 Reduced levels of caspase-3, MDA, 8-OHdG, and TNF-α Promoted the recovery of SOD, catalase, and IL-10 Ameliorated radiation-induced brain injury	[58]
			IVT	Nucleus pulposus cells	iMSCs	miR-105-5p	SIRT6 activation Rejuvenated senescent nucleus pulposus cells and attenuated intervertebral disc degeneration	[59]
	**Telomere ** **attrition**	Pro-	IVT	Peripheral blood mononuclear cells	TRF2-induced BJ-5ta fibroblasts	cfTERRA	Stimulated the transcription of inflammatory cytokine genes (TNFα, IL6, and C-X-C chemokine 10)	[71]
			IVT	Breast cancer cells	Irradiated breast cancer cells	Proteins and RNAs	Reduced telomere length Reduced telomerase activity	[73]
		Anti-	IVT IVV	BM-MSCs Ovariectomized mice MRL/lpr mice (systemic lupus-erythrematosus-like)	Stem cells from human exfoliated deciduous teeth (SHED)	miR-346	Increased Tert mRNA expression and telomerase activity Increased hematopoietic niche formation and osteoblast differentiation Recovered bone volume and alleviate osteoporosis	[74,75]
			IVT IVV	T cells and T cells deficient in telomerase (TERT KO cells)	Autologous-antigen-presenting cells (polymorphonuclear cells)	Telomeric DNA	Increased telomere length by Rad51 recombination factor Increased T cell proliferation rates and delayed senescence, increase in central memory T cells Increased mice survival after viral infection	[78]
			IVV	Old mice	Serum of young mice	Several miRNAs (miR-126b-5p)	Upregulated telomerase-complex-related genes (*Men1* and *Mre11a*) in the liver and lungs	[76]
	**Epigenetic ** **alterations**	Pro-	IVT	Human mononuclear cells from healthy donors	K-562 cell line (lymphoblasts)	NA	Global DNA hypermethylation, including promoters of the tumor suppressor genes P53 and RIZ1, and the upregulation of methyltransferases	[55]
			IVT	HUVECs	Senescent HUVECs	miR-21-5p and miR-217	Downregulation of DNA methyltransferase 1 and SIRT1 Expression of SASP molecules and cell cycle inhibitors	[82]
		Anti-	IVV	Old mice	Young ADSCs	miRNAs	Reduced epigenetic clocks of the liver and kidney Improved renal function and increase healthspan	[77]
	**Loss of ** **proteostasis**	Pro-	IVT		N2a cells (neural-crest-derived cell line)	Amyloid beta peptide (Aβ)	Responsible for the release of Aβ into the extracellular milieu Involved in the pathogenesis of AD	[88]
		Anti-	IVT IVV	Podocytes	ADSCs	miR-486	Promoted autophagy Attenuated diabetic nephropathy	[89]
			IVV	Renal tissue	BM-MSCs	NA	Autophagy induction through the mTOR signaling pathway Attenuated diabetic nephropathy	[90]
			IVT IVV	Hepatic tissue and HSC-T6 cells	ADSCs	miRNA-181-5p	Autophagy activation Prevented liver fibrosis	[91]
			IVT IVV	Cardiomyocytes	BM-MSCs	NA	Autophagy activation via AMPK/mTOR or Akt/mTOR Rescued myocardial ischemia/reperfusion injury	[92]
			IVT	SH-SY5Y cell line (from neuroblastoma cell line)	h-UCMSCs	NA	Induced autophagy in neural tissue Improved Parkinson’s disease features	[93]
			IVT IVV	Neurons under glucose–oxygen deprivation Mice exposed to cerebral ischemia	ADSCs	miR-25-3p	Reduced autophagy and cell death by modulating p53-BNIP3 signaling Reduced infarct size and improve neurological recovery	[94]
***A*** ***N*** ***T*** ***A*** ***G*** ***O*** ***N*** ***I*** ***S*** ***T*** ***I*** ***C***	**Deregulated nutrient ** **sensing**	Pro-	IVT	Young MSCs	Old MSC-EVs	NA	Increased mTOR pathways	
		Anti-	IVV	Old mice	Plasma from young mice	miR-126b-5p and miR-466c-5p	Reduced mTOR and IGF1R levels in the lungs and liver	[76]
			IVV	Old mice	BM-MSCs	NA	Reduced mTOR and IGF1R levels in the parietal cortex Promoted microglial M2 polarization	[101]
			IVT	Cardiomyocytes	BM-MSCs	NA	AMPK activation results in increased autophagy Rescued myocardial ischemia/reperfusion injury	[92]
			IVT	Old MSCs	Young MSCs	miR-188-3p	Rictor targeting downregulates mTOR pathways and increases AMPK pathways. Improved pluripotency of MSCs	[27]
			IVV	Old mice	Plasma from young mice	eNAMPT	Increased NAD+ bioavailability in tissues Increased lifespan and healthspan	[22]
	**Mitochondrial dysfunction**	Pro-	IVT	Old mice		mtDNA	mtDNA in mitovesicles declines with age Cell-free mtDNA causes chronic inflammation	[113]
			IVT	Lung epithelial cells	Idiopathic pulmonary fibrosis—lung fibroblasts	miR-23b-3p, miR-494-3p	Suppressed SIRT3 expression Increased mitochondrial ROS and mitochondrial damage	[114]
		Pro- Anti-	IVT	HeLA cells	Plasma from young and old mice	NA	Improved (young EVs’) or worsened (old EVs’) oxygen consumption rates	[113]
		Anti-	IVT	MDPSCs from old mice	Serum from young and old mice	α-Klotho transcripts	Increased basal oxygen consumption rates Improved mitochondrial ultrastructure	[115]
			IVT	MDPSCs	Oxidatively injured myotubes	NA	Increased basal oxygen consumption rates and spare respiratory capacity	[116]
			IVV IVT	mtDNA-deficient L929 Rho0 cells Mice with multiple sclerosis	NSCs	Functional mitochondria	EV-transferred mitochondria replaced the cell’s own, improving mitochondrial function Increased cell survival When taken up by immune cells, decreased inflammation Ameliorated clinical deficits	[117]
			IVV IVT	Mice with ARDS-like damage Primary human airway epithelial and pulmonary endothelial cells	MSCs	Functional mitochondria	Restored cell barrier integrity and levels of oxidative phosphorylation Restored mitochondrial respiration in lung tissue Reduced lung injury in ARDS	[118]
			IVV IVT	Hypoxia-injured iPSC-derived cardiomyocytes Mice with myocardial infarction	Autologous-stem-cell-derived cardiomyocytes	Functional mitochondria PGC-1α	Improved mitochondrial function Increased mitogenesis Significantly improved post-infarction cardiac function	[119]
			IVV	Mice with acute kidney injury	MSCs	TFAM	Stabilized mtDNA via the formation of the TFAM–mtDNA complex Reversed mtDNA deletion and mitochondrial oxidative phosphorylation defects Attenuated renal damage	[120]
			IVV	Mice with AD	MSCs, SHP2-enriched	2 SHP	Selective induction of mitophagy in neural cells Decreased apoptosis and inflammation Decreased synaptic loss and cognitive decline are reduced	[121]
	**Cellular ** **senescence**	Pro-	IVT	MSCS	Senescent MSCs	miR-31	Inhibited osteogenic differentiation via Frizzled-3 factor knockout Inhibited proliferation	[138]
			IVT IVV	BMSCs	Senescent muscle cells	miR-34a	Senescence induction and decreased SIRT1 expression	[139]
			IVT	HFFF2 human foreskin primary fibroblasts	Senescent HFFF2 expressing an empty vector or oncogenic H-RAS (iC and iRAS cells)	IFITM3	Senescence induction via paracrine transmission	[140]
			IVV	HFFF2 human foreskin primary fibroblasts	Senescent HFFF2 expressing an empty vector or oncogenic H-RAS (iC and iRAS cells)	NA	Senescence induction through the NF-κB/IKK pathway	[141]
		Anti-	IVT	Fibroblasts from old mice or progeroid mice	Fibroblasts from young mice	GSTM-2	Senescence markers decreased (p16, p21, SA-ß-Gal and yH2AX) Reduced ROS and lipid peroxidation levels	[27]
			IVT	Senescent MSCs	MSCs	Peroxiredoxins	Decreased senescence and ROS	[143]
			IVV IVT	Ercc1−/− mice MSCs from old mice Progeroid MDPSCs (Zmpste24−/−)	hESC-derived BM-MSCs	miRNAs	Decreased senescence in vivo in kidney, liver, lung, and brain Decreased senescence in vitro (p16, p21, p53, PTEN, MYC, IL-1, and IL-6) through a possible senomorphic effect (downregulation of SASP factors) Increased the lifespan of Ercc1−/− mice	[144]
			IVT	UV-radiated dermal fibroblasts	h-UCMSCs Dermal fibroblasts	NA	Decreased senescence Promoted the expressions of GPX-1 as well as Col-1 and decreased the expression of MMP-1	[145]
			IVV IVT	Old mice Senescent C2C12 myoblasts	Young ADSCs	miR-125b-5p, miR-let7c-5p, and miR-214-3p	Decreased senescence in vivo in kidney and muscle (laminB1, yH2AX) through a possible senomorphic effect (downregulation of SASP factors) Decreased senescence in vitro (SA-ß-Gal, SASP factors) with no selective increase in apoptosis	[77]
***I*** ***N*** ***T*** ***E*** ***G*** ***R*** ***A*** ***T*** ***I*** ***V*** ***E***	**Stem cell ** **exhaustion**	Anti-	IVT	MSCs from old mice Progeroid MDPSCs	hESC-derived BM-MSCs	miRNAs	Decreased senescence of BM-MSCs Decreased senescence of MDPSCs and increased differentiation	[144]
			IVV	Old mice	hESCs	Proteins modulating anti-aging genes	Promoted proliferation and osteogenic differentiation of BM-MSCs Alleviated age-related bone loss	[34]
			IVV	Old mice	Hypothalamic NSCs from young mice	miRNA	Improved healthspan (physical tests, memory, and socialty) Rescued hypothalamic NSCs’ senescence	[36,155]
			IVV	Old mice	ESCs	SMADs 4-5	Activation of the MYT1–Egln3–Sirt1 axis Improved hNSCs’ stemness	[156]
			IVV	Ovariectomized mice	SHED	miR-346	Increased BM-MSCs’ proliferation and stemness Recovered bone volume and alleviate osteoporosis	[74]
			IVV	Old mice	Young ADSCs	miRNAs	Increased proliferation and reduced fibrosis of renal tubules Improved renal function Higher cross-sectional area of muscle fibers, predominancy of type II fibers, and higher protein content Improved physical condition, decreased frailty scores, and increased healthspan	[77]
			IVT	MDPSCs from old mice	Serum from young and old mice	α-Klotho transcripts	Increased myogenic differentiation potential	[115]
			IVT	Senescent DP-MSCs (passage or hyperoxia pretreatment)	DP-MSCs	miR-302b	Increased expression of the pluripotency factors OCT4, SOX2, KLF4, and cMYC via HIF-1α activation A metabolic shift towards highly glycolytic and low oxidative profiles Recovered stemness	[32]
	**Altered ** **intercellular communication**	Anti-	IVT IVV	Cardiomyocytes, H9c2 myoblasts, cardiac fibroblasts, and HAPI cells	ADSCs	NA	Promoted macrophage M2 polarization and decreased LPS-induced inflammation Attenuated hypoxic damage	[163]
			IVT	Osteoarthritic osteoblasts	ADSCs	NA	Reduced the levels of inflammatory mediators (IL-6 and PGE2)	[164]
			IVV	Intestinal epithelial cells	BM-MSCs	NA	Reduced IL-17A, RORγt, and IL-23	[56]
			IVV	Serum, CNS, and salivary glands	Young mice serum	NA	Reduced the levels of inflammatory mediators (IL-6, IL-1β, and TNF-a) Reduced CD4+IFN-γ+ T cells Reduced CNS-penetrating CD3+ T cells and macrophages, as well as MHC-II expression by microglia Reduced chronic autoimmune predisposition	[165]

## Data Availability

Not applicable.
